# Regulation of *PURA *gene transcription by three promoters generating distinctly spliced 5-prime leaders: a novel means of fine control over tissue specificity and viral signals

**DOI:** 10.1186/1471-2199-11-81

**Published:** 2010-11-09

**Authors:** Margaret J Wortman, Laura K Hanson, Luis Martínez-Sobrido, Ann E Campbell, Jonas A Nance, Adolfo García-Sastre, Edward M Johnson

**Affiliations:** 1 Department of Microbiology and Molecular Cell Biology, Eastern Virginia Medical School, 700 W. Olney Road, Norfolk, VA 23507, USA; 2 Department of Biology, Texas Woman's University, 304 Administration Drive, Denton, TX, 76204, USA; 3 Department of Microbiology and Immunology, University of Rochester, School of Medicine and Dentistry, 601 Elmwood Ave, Rochester, NY 14642, USA; 4 Department of Microbiology, Mount Sinai School of Medicine, One Gustave Levy Place, New York, NY 10029, USA; 5 Department of Medicine, Division of Infectious Diseases, Mount Sinai School of Medicine, One Gustave Levy Place, New York, NY 10029, USA; 6 Global Health and Emerging Pathogens Institute, Mount Sinai School of Medicine, One Gustave Levy Place, New York, NY 10029, USA

## Abstract

**Background:**

Purα is an evolutionarily conserved cellular protein participating in processes of DNA replication, transcription, and RNA transport; all involving binding to nucleic acids and altering conformation and physical positioning. The distinct but related roles of Purα suggest a need for expression regulated differently depending on intracellular and external signals.

**Results:**

Here we report that human *PURA *(*hPURA*) transcription is regulated from three distinct and widely-separated transcription start sites (TSS). Each of these TSS is strongly homologous to a similar site in mouse chromosomal DNA. Transcripts from TSS I and II are characterized by the presence of large and overlapping 5'-UTR introns terminated at the same splice receptor site. Transfection of lung carcinoma cells with wild-type or mutated *hPURA *5' upstream sequences identifies different regulatory elements. TSS III, located within 80 bp of the translational start codon, is upregulated by E2F1, CAAT and NF-Y binding elements. Transcription at TSS II is downregulated through the presence of adjacent consensus binding elements for interferon regulatory factors (IRFs). Chromatin immunoprecipitation reveals that IRF-3 protein binds *hPURA *promoter sequences at TSS II in vivo. By co-transfecting *hPURA *reporter plasmids with expression plasmids for IRF proteins we demonstrate that several IRFs, including IRF-3, down-regulate *PURA *transcription. Infection of NIH 3T3 cells with mouse cytomegalovirus results in a rapid decrease in levels of *mPURA *mRNA and Purα protein. The viral infection alters the degree of splicing of the 5'-UTR introns of TSS II transcripts.

**Conclusions:**

Results provide evidence for a novel mechanism of transcriptional control by multiple promoters used differently in various tissues and cells. Viral infection alters not only the use of *PURA *promoters but also the generation of different non-coding RNAs from 5'-UTRs of the resulting transcripts.

## Background

The Pur protein family of sequence-specific single-stranded nucleic acid-binding proteins is extremely well conserved from bacteria through humans [1]. Human and mouse Purα differ by only 2 amino acids. Human family member, Purα, has a primitive codon usage preference resembling that in bacteria. Purα was first identified due to its ability to bind a purine-rich sequence in an initiation zone of DNA replication upstream of the *c-MYC *gene [2-4]. It was quickly recognized that Purα has a high affinity for single-stranded oligonucleotides, DNA or RNA, with a purine-rich repeat, (GGN)_n _[3]. Purα binds the G-rich strand of double-stranded DNA [3] with the ability to locally separate the bound strands [5-7]. Purα's role in DNA replication has been supported in several laboratories (8-12). Shimotai [8] determined that Purα binds an element within an autonomously replicating sequence (ARS) in the rat aldolase B promoter that is essential for replication. Liu et al. found that Purα and Cyclin A/Cdk2, two proteins essential for the initiation of replication, are simultaneously bound to the replication origin near the c-MYC gene [9]. Purα has also been associated with viral DNA replication. Purα binds the JC viral (JCV) origin of replication and at higher concentrations, inhibits JCV DNA replication [10]. At lower concentrations Purα cooperatively interacts with HIV-1 Tat and large T-antigen to enhance JCV DNA replication [11,12].

Several papers indicate that Purα is multifunctional in cell cycle regulation. During early G1, Purα associates with hypophosphorylated retinoblastoma protein, Rb, from which it is released when Rb becomes phosphorylated in mid to late G1 phase [13]. Purα functions in maintaining the integrity of replicating DNA [14]. Purα remains bound to Cyclin A/Cdk2 at the G2/M boundary [9,15], a checkpoint for replication fidelity. The Cyclin A/Cdk2 complex is important in the repair of double-strand breaks [16]. Wang et al. [14] found that mouse embryo fibroblasts (MEFs) depleted of Purα, have an increased incidence of double-strand breaks when grown in the presence of the DNA replication inhibitor hydroxyurea (HU). Deletions or translocations of *PURA *correlate with the occurrence of myelodysplastic syndrome and its transition to acute myelogenous leukemia [17].

Transcription of a given gene can be initiated at more than one site. Analyses of full-length human cDNAs from a large number of cDNA databases has revealed that human genes, especially those encoding certain proteins expressed in the brain, have multiple putative alternative promoters (PAPs) [18]. On average there are 3.1 PAPs per gene with frequent variation in splicing of the first exon. It is known that Purα is highly expressed in the brain [19] and that in the mouse it has one intron in its 5' untranslated region (5'-UTR). Tissue specificity in the usage of PAPs was observed [18]. Multiple PAPs allow transcription to be regulated by different sets of regulatory factors. In the cases thus far studied, different promoters have generated different coding sequences. We hypothesize that Purα expression is controlled differently depending on various states of cell environment, including growth-altering signals or viral infection and that such control is mediated effectively by independent modes of transcriptional promotion. Here we identify three functionally distinct *PURA *promoters and show that these are utilized differentially in human cell types and that they respond differently to cytomegalovirus infection. While all three promoters generate the same protein coding sequence, multiple levels of control are exerted over the sequences of transcripts produced and over cellular levels of Purα protein.

## Methods

### Cell Culture

NCI-H82 and NCI-H146 small-cell lung carcinoma (SCLC) cell lines were obtained from the American Type Culture Collection (ATCC, Manassas, VA) and were maintained in RPMI medium supplemented with L-glutamine and antibiotics as described by ATCC. NIH 3T3, a mouse embryonic fibroblast derived cell line was maintained in culture and infected with mouse cytomegalovirus (MCMV) as previously described [20].

### RNA Analysis

Total RNA from normal lung tissue and lung adenocarcinoma (prepared by Clontech, Mountain View, CA) was used to analyze *PURA *5'-UTR RNA. C-DNAs were synthesized using the ImProm-II Reverse Transcription System (Promega, Madison, WI) and a primer located 115 nt upstream of the translational start codon. Sequence closer to the translational start codon was avoided because of its high G:C content. *PURA *containing transcripts were amplified with Taq DNA polymerase as described by Promega in a Hybaid OmniGene Temperature Cycler. The initial PCR products were purified on Qiaquick PCR purification columns (Qiagen, Valencia, CA) and the DNA re-amplified with nested primers in a reaction mixture made according to the directions of the Expand HiFidelity PCR System (Roche Applied Science, Indianapolis, IN) but with the additions of 5% DMSO and 1.5 μM betaine. Primers prepared by Invitrogen, (Carlsbad, CA) are described in the text. Positions and sequences are listed in Table 1.

**Table 1:** Primers used in PCR and RT-PCR

Location	Primer	F/R	species	5'base	3'base	5'- to -3'
**TSSI**	0	F	Hs	-6131	-6115	CCCGCAGAGCCTAAAGTGAG

**TSSII**	1	F	Hs	-1454	-1435	GGGAGTGGGATCTCTCCTTG

**TSSII**	2	F	Hs	-1308	-1289	TCTCCTCCATTCTGGGTCCT

**TSSII**	3	R	Hs	-1250	-1268	GGCTAGGGTCTGAATGCTCA

**TSSII**	4	R	Hs	-1201	-1220	GAGCCCAGCCTGAGTTGCTG

**TSSII**	5	F	Hs	-1135	-1115	CAGTCAGTCGTGGTCCCAAGG

**TSSII**	6	F	Hs	-1104	-1086	GCAGGTTTTTGGGTGAGTG

**TSSII**	7	F	Hs	-1038	-1018	GGCAGAGTCGACGGGAACAGT

**TSSII**	8	R	Hs	-1031	-1049	ACTGTTCCCGTCGACTCTGCC

**intron**	9	R	Hs	-913	-933	GTGGCTCTGGCCTCAGATGAT

**intron**	10	R	Hs	-842	-824	GCTAGGTGTCTGCTGCTCC

**intron**	11	R	Hs	-514	-534	AGCACAGAGAGTGGGCCTAAG

**intron**	12	F	Hs	-185	-164	CAGTGCCCTGTTACCGGGTCTC

**TSSII&III**	13	R	Hs	-119	-138	CCAGTCAGCCACTCTCGCGA

**TSSII&III**	14	R	Hs	-115	-134	ACAGCCAGTCAGCCACTCTC

**cds**	15	F	Hs	780	799	CTACAAGGTGTGGGCCAAGT

**cds**	16	R	Hs	952	933	CTTCCTCACCCTGCAGTAGC

						

**TSSII**	Mn1	F	Mm	-1061	-1042	GCAGGTTTTTGGGTGAATGT

**TSSII**	Mn2	F	Mm	-1003	-984	AGCCTACGGGAATAGGCACT

**TSSII**	Mn3	R	Mm	-981	-1000	CGCAGTGCCTATTCCCGTAG

Hs - Homo sapiens; Mm - Mus musculus

All human and mouse primers used in PCR experiments are listed. Their locations upstream from the start codon, direction (F represents the coding strand) and the features within the promoter near their locations are given. (The locations of primers are illustrated for human, Figure 2A and mouse, Figure 8A).

The MTC™ Panel I (Clontech, Mountain View, CA) of first strand cDNAs prepared from poly A+ RNA isolated from eight human tissues was used to quantitate *PURA *transcripts. Real time (RT)-PCR was performed using the ICycler (BioRad, Hercules, CA) with primers described in the text (also see Table 1) and processed using ICycler software. RT-PCR reaction products were routinely analyzed by electrophoresis in 0.8% agarose gels in 40 mM Tris-Acetate, 2 mM disodium EDTA (TAE), pH 8.5, and stained with SYBR Gold nucleic acid gel stain (Molecular Probes, Invitrogen). When reactions resulted in PCR products of < 200 bp, they were also analyzed in 8% polyacrylamide gels in the same buffer.

### mPurα expression in murine cytomegalovirus (MCMV)-infected cells

NIH 3T3 fibroblasts were propagated and infected with wild-type Smith strain MCMV as previously described [20,21]. For the current experiments, cells were infected with a multiplicity of 2 PFU/cell, and infected cell lysates were harvested at 1, 3, 5, and 9 hr post infection. Total cell protein and RNA was isolated using TRI Reagent (Molecular Research Center, Inc., Cincinnati, Ohio) according to manufacturer's instructions. RNA was further processed using RNAeasy columns (Qiagen) with DNase I digestion. Mock-infected NIH 3T3 cells were exposed to the same complete media in which the virus stocks were prepared [21]. The recovered proteins were separated by 8% SDS-PAGE, and analyzed by Western immunoblotting. The anti-Purα monoclonal antibody has been described [22].

### PURA promoter reporter constructs, and transfection

To clone the *PURA *5'-UTR and promoter sequences, DNA isolated from normal lung tissue (Oncomatrix, Inc., San Diego, CA) was used as template in PCR reactions using the Expand Hi-Fidelity PCR System (Roche Applied Science, Indianapolis, IN) with primers chosen to amplify a sequence extending from the ATG translational start codon to a site 2,548 bp upstream (5'-TTTAGGTACCTGCTACTGTTCGAGCCTGG-3'; 5'-TTTAAAGCTTTGATGCTGCGCTC CGCTGC-3', Gene Link, Hawthorne, NY) as above. The amplified sequence was ligated into the KpnI and HindIII sites in the luciferase reporter gene construct, pGL3-Basic Vector (Promega, Madison, WI) to produce pGL3-*PURAPr*. All *PURA *constructs were made using this vector, which places the *PURA *promoter upstream of the luciferase reporter gene. The *PURA *sequence was confirmed by sequencing. The deletion construct pGL3-*PURAPr*Δ(-1514 to -1202) was made by eliminating sequence between the existing restriction sites PacI and BlpI. Other deletion mutants were made by synthesizing short sequences, by PCR amplification, on either side of the intended deletion. The PCR generated fragments were then ligated to nearby sites generated with restriction enzymes. These mutants include pGL3-*PURAPr*Δ (-190 to -85); pGL3-*PURAPr*Δ(-173 to -141); pGL3-*PURAPr*Δ(-141 to -85); and pGL3-*PURAPr*Δ(-141 to -13). All constructs were grown in XL10-Gold Ultracompetent Cells (Stratagene, La Jolla, CA). Plasmids were isolated from bacterial cultures using the EndoFree Maxi Plasmid Purification Kit (Qiagen, Valencia, CA).

SCLC lines NCI-H82 and NCI-146 were transfected with pGL3-*PURAPr *or pGL3-*PURAPr *mutants and the expression plasmid pRL-CMV (Promega, Madison, WI), to determine transfection efficiency, in a medium containing the transfection reagent Fugene (Roche Applied Science) according to manufacturer's directions. Forty-eight hours post-transfection, cells were lysed and processed for analysis using the Dual-Luciferase Reporter Assay System, (Promega, Madison, WI). Light emissions were read in a Turner Designs TD-20/20 Luminometer. Statistical calculations were obtained using GraphPad Prism 4.0 for Mac.

### IRF expression constructs and analyses of IRF effects in transfected cells

The pCAGGs expression vector, which utilizes a β-actin/β-globin hybrid promoter to express mammalian genes [23], was used to generate the pCAGGs GFP expression plasmid by cloning the GFP open reading frame from pEGFP-C1 (Clontech) into the pCAGGs vector. Multicloning sites in the amino- and carboxy-terminal domain of GFP were created to generate NH2 and/or COOH terminal fusions to GFP. Plasmid pEGFP-C1 hIRF-3 has been previously described [24]. Human IRF-5 was amplified by RT-PCR from RNA isolated from human dendritic cells (kindly provided by Dr. Nina Bhardwaj, Departments of Medicine, Pathology, and Dermatology, NYU Medical Center, NYC, NY) and cloned into pCAGGs. The pEGFP-C1 hIRF-5 plasmid was generated by subcloning the hIRF5 ORF into the pEGFP-C1 expression vector. Human IRF-7 was amplified from the plasmid pBS-hIRF-7A (a gift from Dr. Luwen Zhang, Center for Virology, University of Nebraska - Lincoln) and introduced into pCAGGs. pCAGGs IRF-7-GFP was generated by introducing the IRF-7 ORF into pCAGGs GFP. Human IRF-1 and IRF-9 were amplified by RT-PCR from RNA isolated from A549 (lung carcinoma) cells and cloned into pCAGGs. pEGFP-C1 hIRF-1 and hIRF-9 were generated by subcloning from pCAGGs into pEGFP-C1. Primers used to amplify the different IRFs are available upon request from L. Martinez-Sobrido. All constructs were verified by sequencing. The expression of the GFP-tagged interferon regulatory factors was confirmed by fluorescent microscopy and by immunoblotting using a GFP monoclonal antibody (catalog number 632375) from Clontech.

To analyze the effects of IRFs we used a transfection system, previously described [24] employing HEK 293T cells. The reporter plasmids pGL3-*PURAPr *or pGL3-*hPURAPr*Δ(-137 to -85) were cotransfected with GFP-tagged IRF-1, IRF-3, IRF-5, IRF-7, IRF-9 expression plasmids or an empty expression vector using Stratagene's (La Jolla, CA) CaPO_4 _based transfection system. Twenty-four hours post-transfection, expression of the GFP tagged constructs was verified by fluorescence microscopy. Twenty-four hours later, cell lysates were prepared and the activation of the promoters was determined using the Luciferase Reporter Assay System.

### Chromatin Immunoprecipitation (ChIP) of IRF-3 transfected cells

HEK 293T cells were transfected with pEGFP-C1(hIRF-3) or pEGFP-C1 control vector as described. Twenty-four hours later cells were processed for ChIP analysis as previously described [25] using IRF-3 pAb (catalog number 39033; Active Motif, Carlsbad, CA) designed for use in ChIP analyses. RT-PCR was performed on the recovered DNA using primers (see Table 1) chosen to amplify DNA sequences near TSS II and downstream.

## Results

### Three distinct regions for the initiation of human PURA transcription

**Figure 1:**
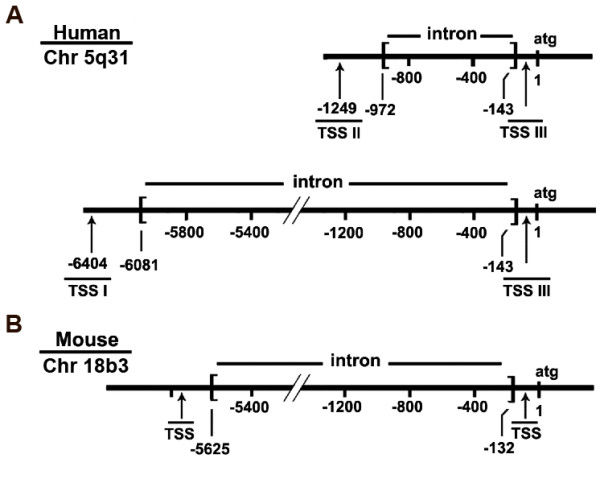
**Comparison of human (A) and mouse (B) PURA transcriptional start sites and 5' untranslated regions. **Three distinct transcriptional start sites (TSSs) initiate transcription of human *PURA*. Human and mouse EST databases were utilized to determine TSSs of *PURA*. Numbering is from the A in the translationally- initiating ATG. A. TSS I - III are shown for humans. B. Two TSSs as determined from ESTs are shown for mouse. The one most distant from ATG has previously been identified [28]. The new mouse TSS is very homologous to human TSS III, which is verified in this report. Human transcripts starting at sites I and II are characterized by introns of dissimilar lengths, as indicated in A. The genomic locations of human and mouse *PURA *are given.

**Figure 2:**
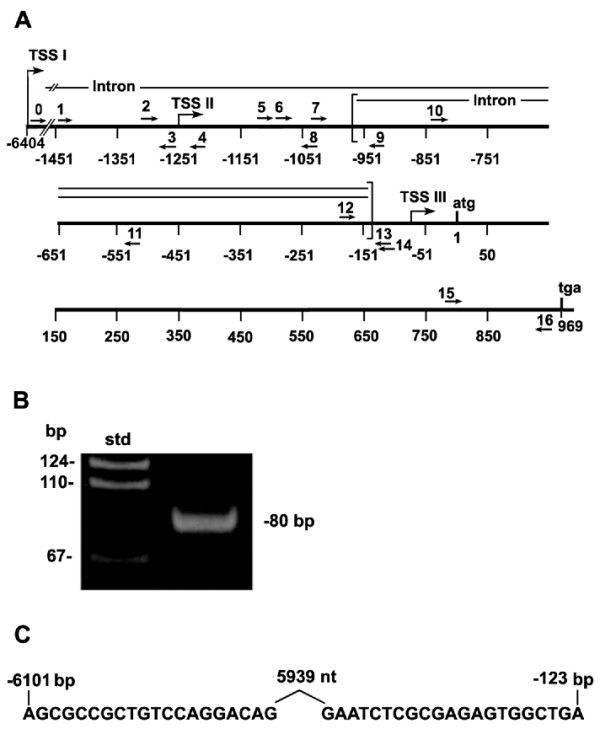
**Human lung TSS I generates a transcript with a 5'-UTR containing an intron of >5 kb. **A. A diagram showing the locations of all human primers used in Figure 2 and in following PCR experiments. B. Reverse transcriptase (rt) -PCR analysis of human lung RNA was performed as described in Methods using primers 0 and 14 (shown in A. and in Table 1). C. DNA sequencing was done to demonstrate the position of a 5939 base intron removed from the transcript. Numbering is as in Figure 1. The sequence shown is a portion of the full 80 bp band of 2B.

Because the Purα protein sequence is so highly conserved [1], we sought to analyze *hPURA *transcriptional regulation for clues as to how the regulation of *PURA *expression can adapt to important cellular processes. As *hPURA *has no TATA-like sequence, there is no single obvious site for the initiation of transcription. To locate established *PURA *transcription start sites (TSSs) we queried both mouse and human EST databases using the BLAST algorithm [26]. Because Purα is very widely distributed in human tissues, the large database of Purα ESTs provides multiple high-quality sequences. Intriguingly, the *PURA *transcripts could be grouped into three distinct lengths, each of which contains multiple closely-spaced 5'ends (Figure 1, Table 1). The discrete nature of these length groups indicates that each corresponds to a separate transcriptional start site (TSS). The 5' ends of the transcripts in TSS I, located most distant from the translational start codon, correspond to the only thus-far-demonstrated mouse TSS [27,28]. The sequence surrounding this TSS is strongly conserved in mouse and humans (Additional file 1, Figure S1) and is, therefore, likely to be a human TSS. We confirmed the utilization of TSS I in humans using *hPURA *promoter specific primers 0 and 14 (Figure 2A) in rt-PCR analysis of human lung RNA. These primers, whose 5' ends are located 6133 and 115 bp respectively, upstream of the translational start codon, yield an 80 bp rt-PCR product (Figure 2B). Sequencing this product confirmed the removal of a 5,939 base intron (Figure 2C). The Group I transcripts are, therefore, characterized by the removal of a large intron of 5,481 bp in mouse and 5,939 bp in human. In both species this intron is completely within the 5'-UTR. It is not extensively analyzed here, as it has already been analyzed in the mouse [28].

The 5'ends of a second transcript size group (those initiated at TSS II) are located approximately 1249 bp upstream of the translational start codon (Figure 1). A comparison of Group II transcripts with genomic DNA reveals that introns of 830 nt are removed from the 5'-UTRs. This observation essentially controls for and dispels the notion that these RACE (rapid amplification of cDNA ends) products are simply randomly terminated. Interestingly, removal of the longer intron in TSS I transcripts eliminates sequences comprising the 5' ends of the Group II transcripts. Splicing in both Group I and Group II utilizes a common splice receptor site (base -143) at the 3' ends (Figure 1).

A third group of transcripts (Group III) have 5' ends located within 80 nt of the translational start codon. This region is particularly rich in GC base pairs (77%). We considered the possibility that these transcripts may result from the failure of reverse transcriptase to transcribe through secondary structures that may form in this GC-rich region.

Analyses which follow in this paper indicate that Group III transcripts are not abortive RACE products or processing products, but that their 5' ends are TSSs under the control of a unique promoter.

### RNA transcripts from human tissues contain PURA 5'-UTRs originating from TSS Group II

**Figure 3:**
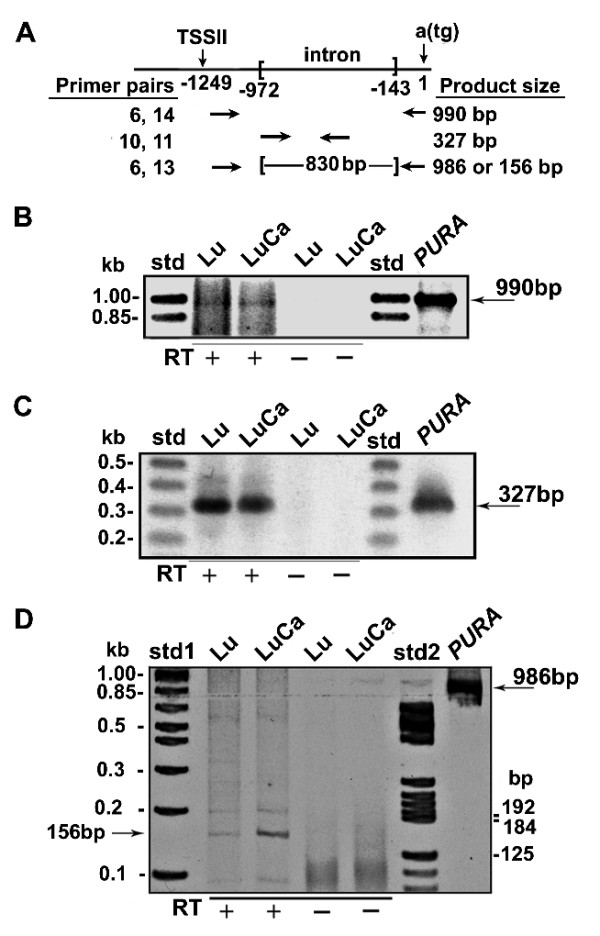
**Human lung and lung adenocarcinoma cells both utilize TSS II and splice out an 830 bp intron. **A. A diagram showing the locations of primers used in PCR in Figure 3 and the size of the resulting PCR products. B. Total RNA from lung (Lu) and adenocarcinoma of the lung (LuCa) was amplified in rt -PCR reactions with primers 6 and 14 yielding a 990 bp band (see Methods). This band is also seen when a DNA sequence containing *PURA *was used as template. These bands are not seen in control reactions, which lacked reverse transcriptase. C. The 990 bp PCR product was further amplified with two nested primers (10 and 11), both within the intron of the transcripts from TSS II, yielding a band of 327 bp. This band is also seen when *PURA *containing DNA is used as template (right lane). D. To demonstrate the removal of the 830 bp intron from the primary transcript, cDNA from lung and adenocarcinoma of the lung was amplified with primer 6 and the nested primer 13 using shortened extension times. A reaction using a *PURA *containing DNA template yielded a 986 bp band (shown at right). The PCR products from Lu and LuCa cDNA contain a band at 156 bp but essentially no band at 986 bp.

We sought to determine whether TSS II, located 1,249 bp upstream of the Purα translational start codon, is utilized similarly in normal human lung tissue and lung adenocarcinoma. *PURA *promoter specific primers (shown in Figure 3A) were used to generate cDNA and then amplify PCR products downstream of the indicated Group II TSS. DNA upstream of the *PURA *translational start codon is particularly rich in G:C base pairs. To avoid an inhibitory effect on reverse transcriptase, we chose to initiate cDNA synthesis from a sequence 115 bp upstream of the *PURA *start codon (Figure 3A). A second primer binding 989 bp further upstream was used to amplify the cDNA in PCR reactions. The resulting PCR products include a band at approximately 990 bp (Figure 3B). Similar reactions that lacked reverse transcriptase did not yield this band. These PCR-DNA products were purified to remove unincorporated oligonucleotides and then reamplified in a second round of PCR reactions with nested primers. The 327 bp product of this reaction can be seen in Figure 3C, first 2 lanes. Genomic DNA incorporated into the construct pGL3-*PURAPr *was utilized in a control reaction. As both primers (10 and 11, Figure 3A) are located within the intron, as indicated by the EST databases, the 327 bp PCR product demonstrates that total RNA as isolated from lung and lung adenocarcinoma contains transcripts which include the Group II intron sequence.

The 5'-UTR of the TSS II transcripts is 830 nt shorter than genomic DNA, raising the possibility of removal of an intron of that length. If RNA isolated from lung tissue or adenocarcinoma includes mRNA with this deletion, a PCR reaction should generate a product 830 bp shorter than control DNA. In genomic DNA the primers (6 and 13) bind at sites 986 bp apart. With the intron removed, the PCR product would be 156 bp. A band of 156 bp is seen in Figure 3D. Both normal lung and adenocarcinoma of the lung contain transcripts with 5' start sites at least 1,100 bp upstream of the translational start codon. The 156 bp PCR-product affirms that an 830 bp intron is removed from both lung and lung adenocarcinoma transcripts (Figure 3D). The intensity of the 156 bp band made from lung adenocarcinoma RNA is clearly greater than the same band from normal lung. By comparing background bands whose concentrations are similar, we can surmise that the greater intensity in the adenocarcinoma is an accurate representation of different levels of spliced transcript in these tissues.

### PURA TSS II constructs identify sequences acting as a promoter in a human cell line

**Figure 4:**
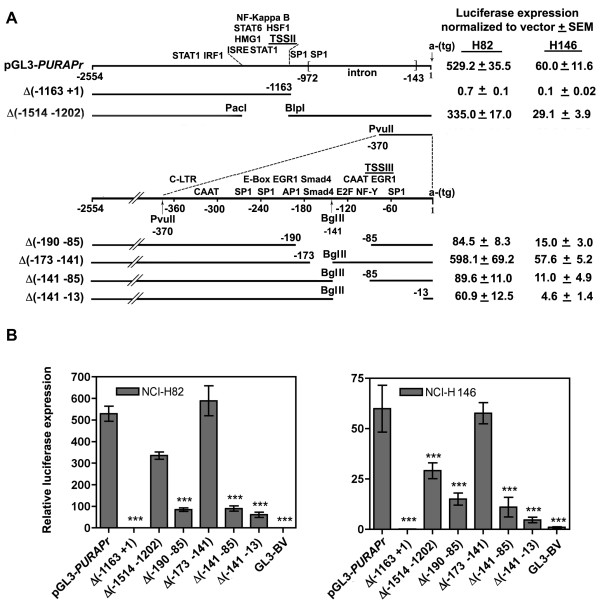
**Transfection of the TSS II PURA promoter and promoter deletion constructs demonstrates that sequences within the promoter are important for PURA transcription. **A. The 2554 bp *PURA *sequence containing TSS II and III as cloned into the pGL3 reporter vector is shown with transcription factor binding elements, MatInspector (Genomatrix). An identified series of deletion mutants is shown below. A, B. NCI- H82 and NCI-H146 cells were cotransfected in triplicate with 1.0 μg of the indicated constructs and 0.1 μg pCMV-RL to correct for transfection efficiency, as described in Methods. Forty-eight hours post-transfection cell lysates were prepared for analysis of luciferase expression (see Methods). Numbers indicate transcription from pGL3-*PURAPr *constructs normalized to transcription from pGL3-BV vector. All statistical analyses were done with two-way ANOVA using the Bonferroni posttest as conducted using Prism 4.0. ***, P < 0.001.

To evaluate the role of *hPURA *promoter sequences on gene expression, we cloned nearly 2,600 bp of genomic DNA from the *PURA *locus, amplified from non-cancerous human lung DNA, into the luciferase expression vector pGL3 forming the construct pGL3-*hPURAPr*. The inserted *PURA *sequence extends upstream from the translational start codon and includes the TSS II located at -1249 bp (Figure 4A). It does not include TSS I (Figure 4A). PGL3-*hPURAPr *was transfected into the small cell lung carcinoma (SCLC) cell lines NCI-H82 and NCI-H146 along with a constitutively expressed Renilla luciferase plasmid. Expression from pGL3-*hPURAPr *increased 529 fold in NCI-H82 and 60 fold in NCI-H146 cells over base levels (Figures 4B), demonstrating that *PURA *is transcribed from TSS II in both cell lines.

### Elements downstream of TSS II positively control its transcription

An examination of the *PURA *promoter by MatInspector http://www.Genomatix.de, a program developed to identify transcription factor binding sites, revealed clusters of transcriptional elements close to both TSS II and TSS III. Several SP1 binding elements called GC boxes, an element frequently associated with transcription of TATA-less promoters, are near both TSSs. TSS II proximal elements are associated with regulating cellular innate immunity, including elements for binding NF-kappaB,, STAT proteins, heat shock proteins, and interferon regulatory factors (IRFs), which are associated with inflammation and response to viruses. This grouping led us to test the extent to which these elements function in transcription. To examine this we developed constructs that deleted selected portions of TSS II or neighboring sequence (Figure 4A). The construct pGL3-*hPURAPr*Δ(-1163 to +1) begins 1400 bp 5' to the Group II TSS and extends 95 bp beyond removing 1154 bp of DNA adjacent to the translational start codon. The removed sequence includes the intron and TSS III elements. This deletion eliminates all transcription in H82 and H146 cells (Figure 4A,B). The construct pGL3-*hPURAPr*Δ (-1514 to -1202) eliminates a region including the TSS II and nearby regulatory elements. Transcription from this construct was reduced to 63% in H82 cells and 48% in H146 (Figure 4A,B). There is clearly a complicated arrangement of stimulatory and inhibitory elements controlling transcription from TSS II. While the decrease in transcription from construct pGL3-*hPURAPr*Δ (-1514 to -1202) demonstrates that elements upstream of TSS II have a positive effect on transcription, the total elimination of transcription from construct pGL3-*hPURAPr*Δ(-1163 to +1) in which the deletion begins 86 bp downstream of TSS II, demonstrates that elements essential for TSS II transcription in SCLC H82 and H146 cells are also located downstream of TSS II. Elements associated with TSS III may thus be important for overall *PURA *transcription in these cells and were thus examined in detail.

### Promoter III contains elements critical for stimulation of PURA transcription

Several transcriptional elements identified by MatInspector are within 300 bp of TSS III. These include a CAAT box, an E-box, and binding sites for E2F1, SP1, NF-Y, and two end-to-end binding sites for Smad4 (Figure 4A). We created the deletion construct pGL3-*hPURAPr*Δ (-190 to -85) to eliminate sites for Smad4, E2F, NF-Y, and the CAAT box. Transcription from this construct was significantly reduced compared to pGL3-*hPURAPr *levels to only 16% in H82 and 25% H146 cells (Figure 4A,B). To further evaluate these elements we divided this deletion between two constructs: pGL3-*hPURAPr*Δ(-173 to -141), which eliminates the two Smad4 binding sites and pGL3-*hPURAPr*Δ(-141 to -85), which eliminates the E2F/CAAT box. While transcription levels of pGL3-*hPURAPr*Δ(-173 to -141) were unchanged statistically in H82 and H146, the 53 bp deletion in pGL3-*hPURAPr*Δ(-141 to -85) significantly decreased transcription to 17% and 18%, respectively. Thus, while the loss of the Smad4 binding sites does not alter *PURA *transcription in this system, the E2F/CAAT region exerts a major influence on *PURA *transcription. The Smad4 sites may be used under conditions that require a direct response to TGF-β stimuli, and such conditions may not have existed here. Extending the deleted sequence to 13 bp upstream of the translational start codon in pGL3-*hPURAPr*Δ(-141 to -13) yields a construct with a similar low level of transcription. This deletion removes a second E2F binding site, which supports the conclusion that E2F is an important factor in regulating transcription from TSS III.

### Differential usage of PURA TSS promoter II in different human tissues

**Figure 5:**
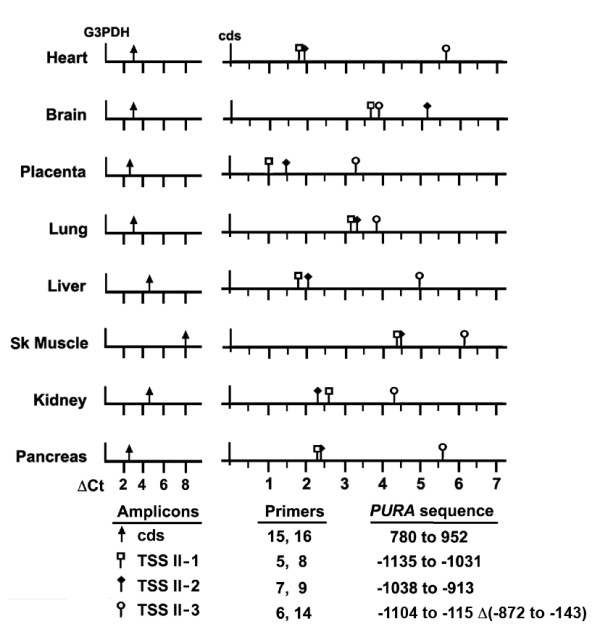
**Relative abundance of PURA transcripts in a panel of eight human tissues. **RT-PCR was performed with 8 human cDNAs using the indicated *PURA *specific primers to amplify sequences downstream of TSS II (TSS II-1, TSS II-2, and TSS II-3) or within the coding sequence (cds) (see Figure 2A) as described in Methods. Primers specific to glyceraldehyde 3-phosphate dehydrogenase (G3PDH) were used to estimate loading. Results are given as the difference in Ct values (ΔCt) determined by RT-PCR for a designated amplicon and a control; ΔCt = Ct (cds) - Ct (G3PDH), left panel; ΔCt = Ct (TSSII-1) or Ct (TSSII-2) or Ct (TSSII-3) - Ct (cds), right panel. Note that a higher Ct indicates a lower level of transcript by log to the base 2. Ct values should be seen as indicating the relative abundance of cDNA amplified by each primer pair and not a quantitative estimate of the difference in abundance of the cDNA sequences amplified in a particular tissue.

Our results raise the important question of whether or not utilization of *PURA *varies in different tissues. To answer this, we determined the relative quantity of *PURA *transcript from the *PURA *coding sequence (cds-RNA) compared to transcript for a control RNA, G3PDH, in 8 human tissues by amplifying in RT-PCR, cDNA prepared for each tissue (Figure 5, left). The ΔCts shown represent relative change. Note, that in Figure 5 each higher Ct indicates a decrease in RNA. In heart, brain, lung, placenta, and pancreas, the ΔCts of *PURA *cds-RNA compared to G3PDH are quite similar. The levels in two tissues, liver and kidney decrease by approximately 2Ct (4-fold). Skeletal muscle appears to have a relatively small amount of *PURA *compared to G3PDH (ΔCts = 7.9), although this large difference may actually reflect the relatively large amount of G3PDH transcript found in skeletal muscle rather than a decreased amount of *PURA*.

To assess the use of TSS II, the relative quantities of 3 sequences within the TSS II transcript and of the *PURA *coding sequence RNA (cds) are compared schematically for each tissue (Figure 5, right). *PURA *cds RNA may include transcript originating from any of the three TSSs and represents the total translatable transcript within the cell. In contrast, the sequence TSS II-1, amplified by primers 5 and 8, is located 115 bp downstream of TSS II but upstream of the intron (see Figure 2A). All transcripts originating from TSS II should have this sequence. TSS II-1 would not be found in transcript originating from TSS I as it is within the intron removed from TSS I transcripts or in TSS III transcripts which originate downstream. The relative abundance of TSS II-1 compared to cds is, therefore, an indication of the relative amount of transcript originating at this promoter within each tissue. TSS II-1 is utilized most often in placenta although the decrease in heart and liver is less than 2-fold. In other tissues brain, lung, and skeletal muscle, the decrease in abundance of TSS II-1 is at least 5-fold indicating that, in those tissues, TSS I or III yield more cds transcript. Two other sets of primer pairs were used to evaluate the frequency at which the intron is removed from the TSS II transcript. The sequence TSS II-2 spans the intron donor site. TSS II-3, amplified by primers 6 and 14, yields a 141 bp product only when the intron is removed. It is interesting to note that Ct values for amplicon TSS II-2 are nearly equal to those of TSS II-1 in heart, lung, liver, skeletal muscle, kidney and pancreas. Only in brain is the intron removed more frequently than not. The observation that the TSS II intron is removed from many brain transcripts indicates that these rt-PCR products are representative of the RNA in these tissues, and not contaminating DNA. We may thus conclude that the TSS II transcript is both produced and processed differently in different tissues.

### Interferon regulatory factors IRF-3, IRF-5, and IRF-7 reduce PURA transcription

**Figure 6:**
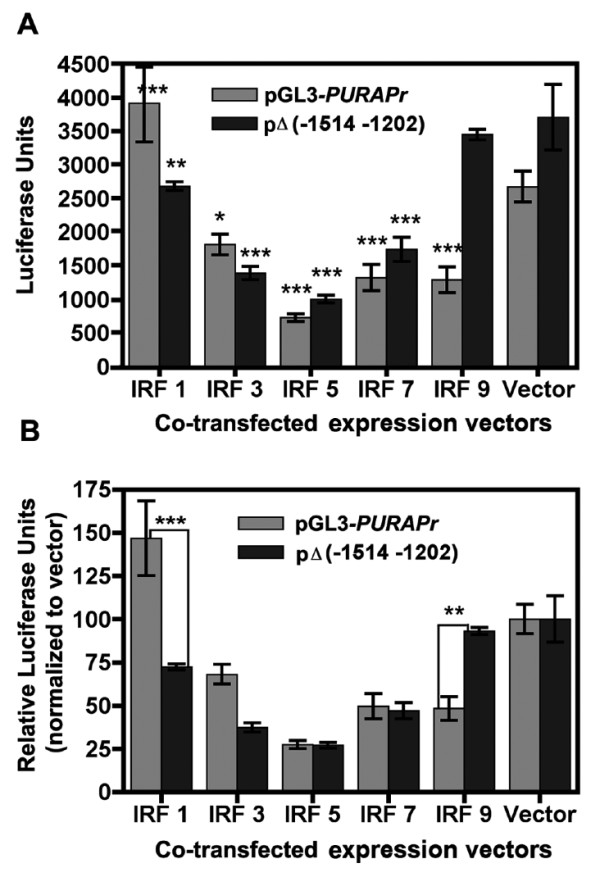
**Transcription from PURA TSS II is decreased when cotransfected with IRF expression plasmids. **A. HEK 293T cells were transfected with 0.5 μg of *PURA *reporter plasmid, and 4 μg of the expression plasmids pEGFP-C1 (hIRF-1, hIRF-3, hIRF-5, hIRF-7, or hIRF-9) or the control plasmid pEGFP-C1. At twenty-four hours post-transfection cells were viewed by fluorescent microscopy for the expression of GFP. At forty-eight hours post-transfection cells were lysed and analyzed for promoter expression as described in Methods. Asterisks indicate a significant difference between transcription from pGL3-*hPURAPr *and pGL3-*hPURAPr*Δ((-1514 -1202) in the presence of the overexpressed IRF protein compared to vector. B. The transcription of pGL3-*hPURAPr *and pGL3-*hPURAPr*Δ((-1514 -1202) in the presence of the overexpressed IRF proteins is normalized to their transcription in the presence of vector, which is set at 100. Asterisks indicate a significant difference between pGL3-*hPURAPr *and the deletion construct. All statistical analyses were done with two-way ANOVA using the Bonferroni posttest. ***, P, < 0.001; **, P < 0.01; *, P < 0.05 as conducted using Prism 4.0.

*PURA *TSS II is near several DNA elements that potentially, bind proteins generated and/or activated by an innate immune response to viral infection. We therefore determined whether or not interferon regulatory factor (IRF) proteins, frequently produced or activated as a result of viral infection, might directly or indirectly alter use of *PURA *promoters. To answer this question we co-transfected HEK 293T cells with GFP-tagged IRF expression plasmids and the *PURA *reporter constructs pGL3-*PURAPr *or pGL3-*PURAPr*Δ(-1514 to -1202) which lacks sequence near TSS II but retains IRF-1 and STAT1 binding sites. The GFP-tagged IRF plasmids overexpress the IRF-GFP proteins, as seen at 24 hr by the large amount of GFP contained within the cells when viewed by fluorescent microscopy. Three of the expressed proteins IRF-3, IRF-5, and IRF-7, reduced transcription to between 67% and 17% of control levels in both constructs (Figure 6A). The observation that pGL3-*PURAPr *and pGL3-*PURAPr*Δ(-1514 to -1202) in the presence of IRF-5 and IRF-7 experienced similar decreases in transcription (Figure 6B) suggests that while IRFs 5 and 7 may act at elements near TSS II including ISRE, an IRF consensus binding element [29], they may act through other pathways, such as binding elements distal to TSS II or indirectly though interaction with cellular proteins. In contrast, IRF-1 increases pGL3-*hPURAPr *while slightly decreasing pGL3-*hPURAPr*Δ(-1514 to -1202) and IRF-9 suppresses pGL3-*hPURAPr *while having no affect pGL3-*hPURAPr*Δ(-1514 to -1202). IRF-1 and IRF-9 are therefore the factors most likely to be acting primarily at elements near TSS II. When pGL3-*hPURAPr*Δ(-1514 to -1202) is cotransfected with pEGFP-C1, the control expression vector, there is an apparent increase, though not statistically significant, in transcription compared to pGL3-*hPURAPr*. This contrasts to the decrease seen following the transfection of pGL3-*hPURAPr*Δ(-1514 to -1202) compared to pGL3-*hPURAPr *into NIH-H82 and NIH-H146 cells (Figure 4B). Differences in these cell types may affect their ability to transcribe from the *PURA *promoters. This will be considered further in the Discussion.

### IRF-3 binds to the TSS II region within the PURA promoter

**Figure 7:**
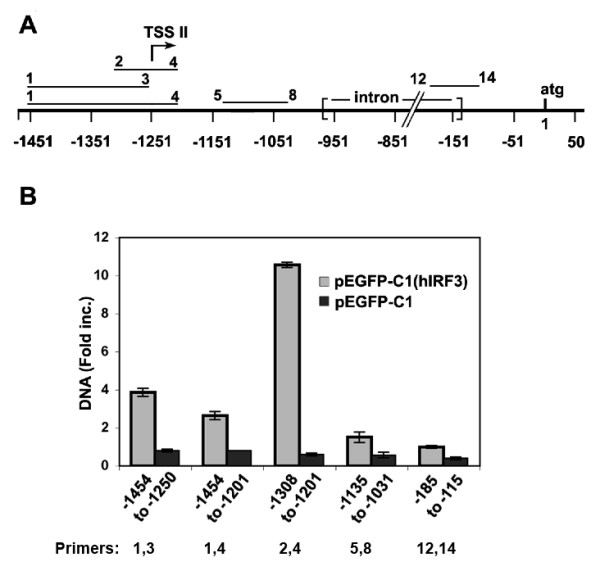
**Chromatin-immunoprecipitation (ChIP) analysis of HEK 293T cells demonstrates that specific sequences near TSS II bind IRF-3. HEK 293T cells were transfected with pEGFP-C1 (hIRF-3) to overexpress IRF-3 or pEGFP-C1 control vector. **After 48 hr cells were fixed as described in Methods and processed for ChIP analysis. Antibody to IRF-3 or rabbit (Rb) IgG was used to precipitate chromatin-bound IRF-3. A. DNA sequences amplified near TSS II and downstream (primers indicated above bars and given in Table 1). Negative numbers indicate base pairs upstream of the start codon. B. Quantities of precipitated DNA from cells transfected with pEGFP-C1(hIRF3) or pEGFP-C1 were determined by RT-PCR. The DNA precipitated with anti-IRF-3 antibody was compared to similar DNA precipitated with Rb IgG, which was considered baseline. Primers for each amplified sequence are given below.

Although IRF-3 clearly affects sites distant to TSS II (Figure 6), because IRF-3 has a distinct inhibitory effect on *PURA *expression, we wanted to examine more specifically its effects at TSS II. IRF-3 is normally found in the cytoplasm of many cell types in an inactive form. Inducible phosphorylation of IRF-3 allows it to form homodimers and heterodimers with IRF-7, which are then transported into the nucleus. In the active (dimerized) state IRF-3 binds to IRF- specific elements in the DNA. To determine if a DNA sequence near TSS II is selectively bound by IRF-3, we transfected HEK 293T cells with the expression plasmid pEGFP-C1-hIRF-3 or with a control vector pEGFP-C1. Although IRF-3 is normally present in the cytoplasm, the large increase in IRF3 would likely facilitate its transcriptional effects. Twenty-four hours after transfection the cells were processed for ChIP analyses. Immunoprecipitations were carried out with IRF-3 pAb (ActiveMotif) which recognizes chromatin bound IRF-3, or with rabbit IgG. Primer sets (Figure 7A) were chosen to amplify in RT-PCR, five sequences located near or downstream of the TSS II promoter. Enhanced binding of IRF-3 was found at four of the five sequences tested (Figure 7B). Binding increased 10.5 fold over control in a 108 bp sequence (primers 2,4) spanning TSS II. Regions just upstream of TSS II (primers 1,3) or downstream of the TSS II (primers 5,8) were amplified to a smaller extent, 3.9 and 1.5 fold respectively. A sequence (primers 12,14) within the TSS III promoter was amplified only to control levels. The binding data indicate that sequences close to TSS II are targeted by IRF-3.

### MCMV infection results in a transient decrease in transcript originating from TSS II

**Figure 8:**
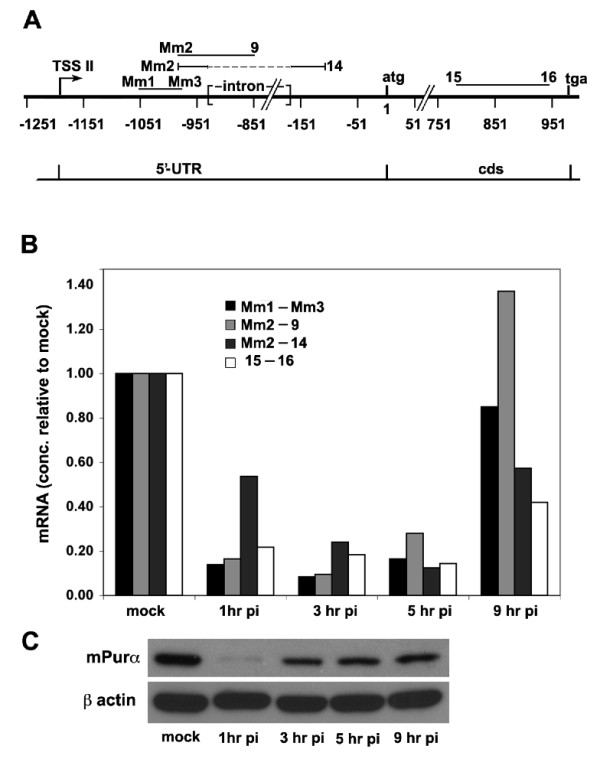
**MCMV infection suppresses PURA transcription and reduces levels of Purα protein. **A. The locations within the *PURA *gene of four sequences amplified by the indicated primers (Table 1). B. The relative concentrations of transcript downstream of TSS II in MCMV infected NIH-3T3 cells as determined by rt-PCR are shown relative to concentrations of transcript in mock treated cells (see Methods). C. Proteins, isolated from the MCMV infected 3T3 cell lysates used in panel B, were assayed for Purα by immunobloting. β actin was used as a loading control.

The decrease in expression of pGL3-*PURA *in the presence of overexpressed IRF-3, IRF-5, and IRF-7 (Figure 6) and the specific binding of IRF-3 to sequence near TSS II (Figure 7) led us to investigate whether or not viral infection could affect *PURA *transcription. We tested this by infecting 3T3 cells with MCMV, a virus which activates IRF-3 upon virion binding to the cell [30]. The similarity of mouse and human Purα and the *PURA *promoter sequence (Additional file 1, Figure S1) render it likely that the response to MCMV infection by mouse cells would be similar to that of human cells. Three sequences within the transcript downstream of TSS II and one sequence within the *PURA *coding transcript were chosen for analysis by rt-PCR. The resulting amplicons are designated by the primers used in their synthesis (Figure 8A, Table 1). The considerable homology in mouse and human *PURA *DNA permitted the use of primers 9, 14, 15, and 16 in PCR analyses with cDNA from both species. The sequence Mm1-Mm3 is downstream from the mouse TSS II and ends upstream of the intron. All transcripts originating from TSS II should, therefore include Mm1-Mm3. The quantity of this sequence drops rapidly during the first hour of infection (Figure 8B); a time when viral particles adhere to cellular receptors and begin entering the cell. Levels of Mm1-Mm3 transcript remain low recovering to the level of mock treated cells by 9 hours. Changes in the relative quantities of amplicons Mm2-9 and Mm2-14 are indicative of changes in the processing of the TSS II transcript. Mm2-9 straddles the intron donor site and is a measure of unprocessed transcript, i.e., transcript retaining the intron. Mm2-14 is amplified with the same 5' primer as Mm2-9 but the 3' primer is located beyond the intron receptor site. Although the Mm2 and Mm14 primers should yield amplicons of two sizes; a 898 bp amplicon from the unspliced transcript and a 102 bp amplicon if the intron is removed, in practice the product is entirely the shorter spliced product, as can be seen following their separation on a TAE acrylamide gel (data not shown). The relative quantity of transcript Mm2-9 drops quickly following infection, a decrease that is similar to the decrease in Mm1-Mm3, but recovers rapidly. At 9 hr pi the level of Mm2-9 exceeds that of mock. In comparison levels of Mm2-14 drop gradually until 5 hr pi, which may reflect a slower turnover of this transcript. Levels of Mm2-14 then recover to levels, which at 9 hr pi are lower than mock. Primers 15 and 16 are located within the *PURA *protein coding sequence. Transcription from all three start sites, TSS I, TSS II, and TSS III, could extend through sequence 15-16. Amplicon 15-16, cds, is therefore a measure of all *PURA *coding transcript in the cell. The level of cds transcript drops dramatically by 1 hr pi and is only partially recovered at 9 hr pi. This large drop in cds transcript implies that synthesis of Purα protein would be reduced significantly soon after MCMV infection.

### Purα levels drop dramatically following MCMV infection

Decreasing levels of *PURA *transcript, particularly of transcript within the coding sequence, raised the question of whether or not Purα protein levels also decrease following MCMV infection. Western blot analysis of proteins isolated from MCMV infected 3T3 cells at 1 hr, 3 hr, 5 hr, and 9 hr pi, and from mock infected cells shows that Purα levels drop dramatically shortly after infection (Figure 8C). At later times Purα levels increase but continue to remain below mock levels during the course of the experiment.

The decrease in Purα levels following MCMV infection requires both the degradation of existing Purα protein and the discontinuation of new synthesis. The rapid decrease in cds transcript (15-16) (Figure 8B) as well as transcript originating from TSS II (Mm1-Mm3) suggests a coordination in the processes of inhibiting transcription from TSS II and of decreasing levels of Purα protein.

## Discussion

These results reveal a novel mechanism for transcriptional regulation of the *PURA *gene that allows separation of promoter controls for cell growth signals and signals for response to viral infection. *PURA *transcription is initiated at three separate start sites that generate distinct transcripts represented in EST databases. Our PCR amplification of transcripts from tissues and cell lines confirms initiation at each of these sites and reveals that each of the three transcripts is differently spliced. Promoter usage and alternative splicing differs among 8 different human tissues examined. Homologies between these promoters in human and mouse and the presence of three mouse EST transcripts similar to the human ones, render it likely that *PURA *transcriptional control is similar in both human and mouse. Human *PURA *TSS I is furthest upstream of the translational start site. Because its promoter is very homologous to a previously characterized mouse promoter [28], we have not further studied it here. In the mouse, this TSS I promoter is notably subject to feedback regulation by the Purα protein [28]. Human TSS II is adjacent to an intriguing cluster of elements potentially involved in an innate immune response to viral infection. We have thus focused here on characterization of promoters II and III. The results obtained are surprising and raise important questions potentially relevant to any gene regulated by multiple promoters. We address some of those questions here.

Clearly, higher eukaryotes have evolved a transcriptional control mechanism, conserved between human and mouse, whereby three different promoters generate transcripts specifying the Purα protein. What evolutionary advantage could that confer? One advantage could be that the three transcripts are utilized differently to express Purα protein. This type of regulation could be similar to that observed in polyomaviruses SV40 and JCV, in which the gene encoding large T-antigen is under control of multiple promoters. In those cases the different transcripts are translated at different rates [31-33]. Another potential advantage could be that the different *PURA *RNAs have specific functional properties. In the rapidly developing field of regulatory RNA, non-coding RNA, often derived from introns in 5'-UTRs, plays an important regulatory role in mRNA translation. It is conceivable that the primary aim of the *PURA *cellular transcriptional manipulation is to generate different non-coding RNA species.

Several viruses are reported to co-opt functions of Purα [12,34]. Is the downregulation of Purα upon MCMV infection a cellular innate immune response, or is it an aspect of viral co-opting of a cellular process? In known instances where Purα function is co-opted by viruses, e.g., upon infection with HIV-1 or JC virus [1,35], viral proteins bind to and alter the function of Purα. That may not be the case regarding certain aspects of the cellular *PURA *response to MCMV. The most dramatic decrease in *PURA *expression occurs at a time when a minimal number of MCMV proteins (immediate early proteins) are synthesized. It remains to be determined whether later aspects of the cellular response to MCMV involve co-opting of Purα functions.

The NIH 3T3 cellular response to MCMV infection involves a very rapid loss of *PURA *mRNA and Purα protein. What is the advantage of such a rapid response, and how is it achieved? Purα is a highly conserved protein that clearly plays a vital role in cell survival [1]. Redundancy of Pur family members is further evidence for the essential nature of this family [19,36]. The protective role of Purα against oncogenic transformation is well documented [13,15,17,37], and it involves rapid changes in intracellular levels during the cell cycle [13,15,37]. Therefore, in order to either modulate or co-opt Purα function, rapid changes in the protein must ensue. It should be noted that the protein Rb is rapidly degraded in response to CMV infection [38,39]. Rb is a well-known binding partner of Purα [13,34,37], and the two act together to regulate the cell cycle. It is thus conceivable that if Rb is subject to proteolysis, its binding partner Purα could be exposed to a similar process of degradation.

The rapid and nearly complete loss of Purα from 3T3 cells within one hour of MCMV infection is intriguing. MCMV could initiate signaling pathways shortly after binding to receptors on the cellular membrane. The integrin receptors are a known point of attachment for HCMV [40]. The pathways may work through proteins, which are constitutively present in the cytoplasm and are integral to the innate immune response. These include proteins such as IRF-3, which becomes phosphorylated and thus activated upon virion binding to the cell [30]. We have shown that IRF-3 binds the *PURA *promoter and down-regulates *PURA *transcription. Regulatory tegument proteins which are transported into the cytoplasm on infection may also be involved [38]. The rapid decrease in levels of both *PURA *mRNA and Purα protein would most likely involve the degradation of both. To reach low levels within one hour, pre-existing *PURA *RNA and protein would need to be destroyed. A transcriptional response to interferon may not be involved. These findings are consistent with microarray data indicating that HCMV infection of monocytes led to a decrease in *PURA *mRNA at immediate early times during infection [41].

The significance in the decrease of Purα should be considered while keeping in mind what is known about Purα levels during the cell cycle. Purα levels fluctuate and reach their lowest level at the onset of S-phase [37]. Elevated levels of Purα in NIH 3T3 cells delay entry into and progression through S-phase [15,42]. It has been reported that MCMV infection alters the cell cycle causing rapid progression toward the G1/S boundary [43,44]. Thus CMV may have evolved a mechanism to decrease Purα levels in order to further a viral propensity to replicate.

There are multiple processes involved in the *PURA *response to MCMV infection. Following the initial degradation of both Purα and *PURA *mRNA, there is a subsequent rapid [40] partial recovery of Purα intracellular levels. This could involve increased translation of existing *PURA *mRNA because at this time levels of such mRNA are still decreasing (Figure 8). It is notable that levels of Purα protein increase modestly from 3 hrs through 9 hrs post infection, although levels of its mRNA are decreasing until at least 5 hrs. It is during this time that an interferon response to viral mRNA would be expected. Levels of Pur mRNA and protein never recover to mock, i.e., uninfected, levels during this time. Thus, the decrease in overall levels of Purα in response to MCMV infection is consistent with our data indicating that IRF proteins repress *PURA *gene transcription. Experiments beyond the scope of this paper will be necessary to fully comprehend the multiple components of *PURA *response to viral infection.

The three *PURA *transcriptional sites identified here as TSS I, II, and III are approximately 6,404 bp, 1,249 bp, and 80 bp upstream from the translational start codon, respectively. Transcripts from TSSs I and II are characterized by the removal of introns of distinctive lengths: 5,939 nt from human TSS I transcripts and 830 nt from TSS II transcripts. No intron is removed from TSS III transcripts or from the *PURA *coding sequence. The question posed is whether these three sites are regulated by different transcriptional elements and with factors associated with different cellular processes. The role of the Group II TSS is very intriguing. An analysis of transcription from this site in 8 human tissues revealed that it contributes to the total *PURA *transcript differently in these tissues. Surprisingly, the intron is frequently not removed from the transcript, and the amount of intron splicing varies with the different tissues. Only in brain tissue is the removal of the intron the usual means of processing. Intron processing also varies between normal and cancerous tissue as seen when comparing lung total RNA to RNA from lung adenocarinoma (Figure 3). While intron RNA is readily detectable in both tissues, adenocarcinoma has a distinctly greater concentration of spliced transcript.

An inspection of potential regulatory elements lying close to TSS II reveals clusters of elements for binding heat shock factor I and factors associated with viral infection, such as interferon, IRF-1, NF-kappa B and the element ISRE which binds the IRF transcription factors. Considering that Purα is required in many processes involving ss-RNA or DNA, the presence of these binding sites suggests that the cell may actually optimize survival during viral infection by regulating the availability of Purα. The ability of the IRFs 3, 5, and 7 to repress transcription from pGL3-*hPURAPr *to as low as 27% of control, supports this hypothesis although these IRFs did not exclusively act near TSS II. IRF-9 appears to act primarily at the TSS II promoter (Figure 6). Using ChIP analysis, we demonstrate that IRF-3 binds a specific DNA site within the TSS II promoter. The ability of IRF-3, usually a positive transcriptional regulator, to down-regulate gene expression has previously been reported by Grandvaux et al. [45].

Analysis of the total RNA recovered from MCMV infected 3T3 cells demonstrates that *PURA *transcription is altered in response to viral infection. Strikingly, there is a significant decrease in the processing of TSS II transcript, which results in a large increase in the amount of transcript from which the intron has not been removed. The binding of IRF-3 and the large number of potential binding elements near TSS II suggest that this promoter is regulated by the interferon directed innate immune response to viral infection. The mechanism of the altered splicing in this case is unknown, but the overall effect is similar to that reported for splicing of various brain transcripts from genes with alternate promoters [18]. Detailing the mechanism of promoter-specific altered mRNA splicing, beyond the scope of this study, will be an important future research subject.

The analyses of transcription from *PURA *TSS II in the human tissue panel and in cells transfected in culture showed that differing cellular environments result in variable amounts of transcription. Moreover, different portions of the promoter sequence can affect transcription differently in various cell types. This is seen when comparing transcription from pGL3-*hPURAPr *Δ(-1514 -1202) in Figure 4 where this deletion mutant gives a significant reduction in transcription relative to control vector, whereas in Figure 6, pGL3-*hPURAPr *Δ(-1514 -1202) yields an increase, not statistically significant, in transcription. It is notable that Figure 4 was done with small-cell lung carcinoma cell lines, each of which highly overexpresses c-MYC [46], and that Figure 6 used HEK 293T cells, which express SV40 large T-antigen (American Type Culture Collection). Each of those proteins are responsible for multiple cellular changes that can have different transactivational effects, including epigenetic changes in DNA methylation or histone modifications that can directly affect transcription of *PURA *and the deletion mutants [47]. These various epigenetic changes affect chromatin conformation causing sequence to vary in its availability to transactivational factors. While elements typically bind factors to facilitate transcription, a different placement of elements could bind the same factors in an arrangement that results in steric hindrance and the suppression of transcription. In this way the IRFs might be used to deny infecting virions cellular proteins that are essential for their replication.

## Conclusions

In summary, we report that a single *PURA *gene has three distinct promoters regulated by factors associated with different cellular processes including cell growth and the innate immune response. Two of the promoters yield long transcripts from which different large introns are removed. The intron of the 5'UTR from TSS II is spliced to different degrees in different tissues. IRF-3, 5, and 7 proteins suppress transcription from TSS II. Following infection by MCMV, the level of *PURA *transcript from TSS II declines rapidly with a corresponding decrease in level of Purα protein.

## Authors' contributions

MJW and EMJ developed the basic experimental design and wrote the manuscript. LM-S and LH devised additional experiments. MW, and LM-S designed and cloned DNA constructs. MW, JAN, LM-S, and LH performed experiments. LH, AEC, MW, AG-S, and EMJ analyzed the data. All authors read and approved the final manuscript.

## Supplementary Material

Additional file 1 **Figure S1. The high level of homology of PURA sequence near TSSI (left) and TSSII (right) in human and mouse genomic DNA. **Sequences were aligned using CLUSTALW multiple alignment, Pole BioInformatique Lyonnais. Red lettering and asterisks indicate homologous sequence. Bent arrows indicate transcriptional start points as identified in EST databases. Click here for file
