# Supplementary figures and images for: Regulation of PURA gene transcription by three promoters generating distinctly spliced 5-prime leaders: a novel means of fine control over tissue specificity and viral signals
Source: BMC Mol Biol. 2010 Nov 9;11:81. doi: 10.1186/1471-2199-11-81 (PMC2992531; doi:10.1186/1471-2199-11-81)

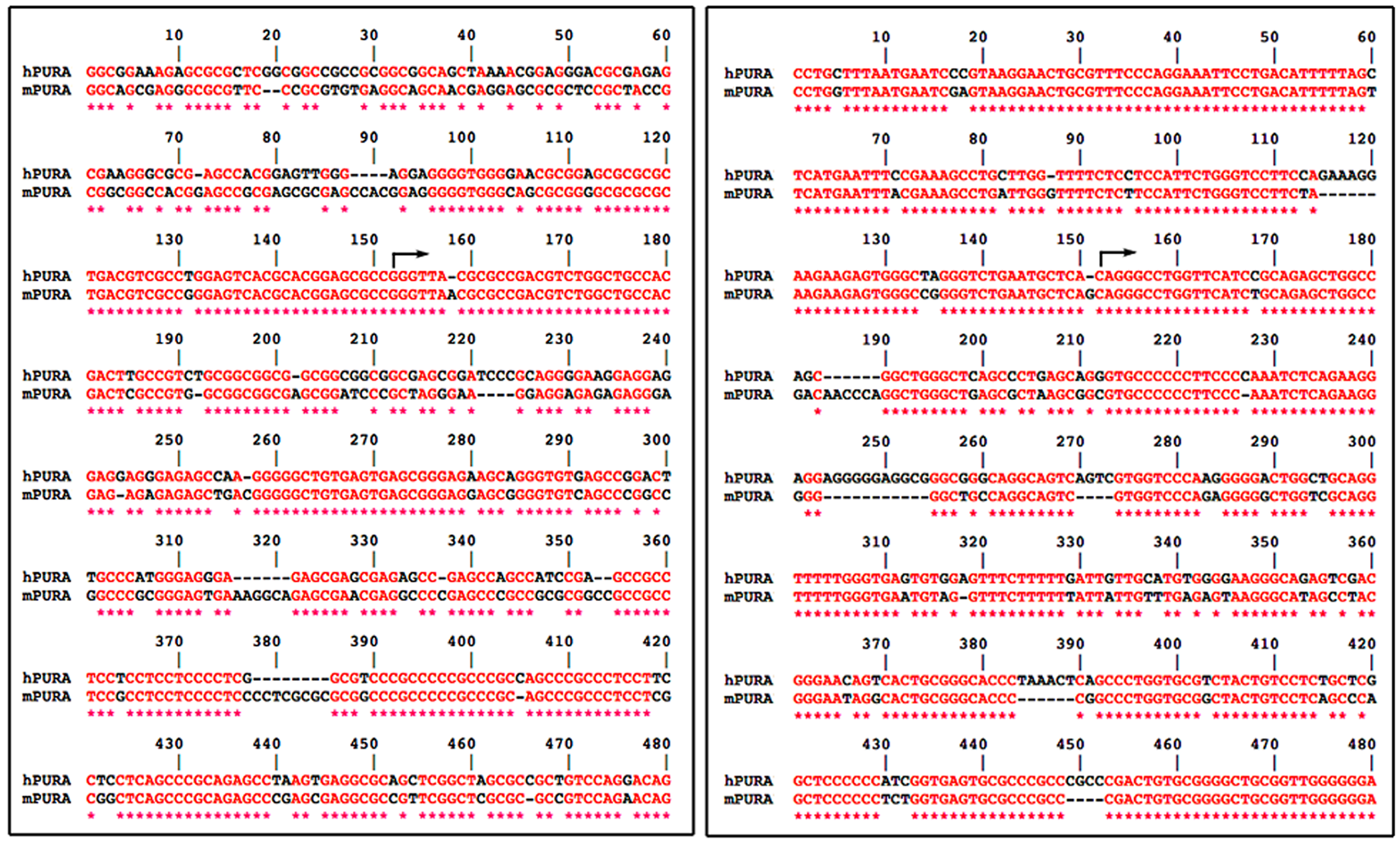

Supplement: Additional file 1 — Figure S1. The high level of homology of PURA sequence near TSSI (left) and TSSII (right) in human and mouse genomic DNA. Sequences were aligned using CLUSTALW multiple alignment, Pole BioInformatique Lyonnais. Red lettering and asterisks indicate homologous sequence. Bent arrows indicate transcriptional start points as identified in EST databases. [file 1471-2199-11-81-S1.TIFF]
